# Building a Resilience Ecosystem to Improve Employee Mental Health and Wellbeing in Canadian High-Stress Low-Control Occupations

**DOI:** 10.3390/ijerph23050669

**Published:** 2026-05-19

**Authors:** Gregory S. Anderson, Yan Song, Rosemary Ricciardelli, Joy C. MacDermid, Heidi Cramm, Deborah Norris, R. Nicholas Carleton

**Affiliations:** 1 Faculty of Science, Thompson Rovers University, Kamloops, BC V2C 0C8, Canada; 2 Marine Institute, Memorial University of Newfoundland, St. John’s, NL A1C 5C4, Canada; 3 School of Physical Therapy, Western University, London, ON N6A 3K7, Canada; 4 School of Rehabilitation Therapy, Queen’s University, Kingston, ON K7L 3N6, Canada; 5 Department of Aging & Family Science, Mount Saint Vincent University, Halifax, NS B3M 2J6, Canada; 6 Department of Psychology, University of Regina, Regina, SK S4S0A2, Canada

**Keywords:** resilience, resilience theory, ecological theory, public safety, occupational health, implementation, mental health

## Abstract

**Highlights:**

**Public health relevance—How does this work relate to a public health issue?**

- Public safety professionals have high rates of post-traumatic stress injury.
- Findings underscore the value of implementing sustained, tailored mental health strategies that include a broader conceptualization of resilience that recognizes factors beyond the individual (including family and workplace), and those guided by recognized frameworks such as the Canadian Standard for Psychological Health and Safety in the Workplace.

**Public health significance—Why is this work of significance to public health?**

- Higher levels of work engagement and resilience were associated with lower extended health costs, suggesting potential pathways toward enhanced performance and reduced psychological service expenditures.
- Psychological stress within the organization is a key driver of health benefit utilization, as higher levels of perceived stress were strongly associated with increased extended health costs.

**Public health implications—What are the key implications or messages for practitioners, policy makers and/or researchers in public health?**

- A National Standard for Psychological Health and Safety in the Workplace can serve as a framework for improving workplace health and safety when integrated with multi-modal action plans and structured resilience programs.
- Fundamental to the longitudinal success of implemented programs is a commitment to Integrated Knowledge Transfer addressing knowledge users’ real-world problems through the co-creation of knowledge and its application.

**Abstract:**

In response to inherent occupational and operational stress in public safety personnel (PSP), multiple policies and interventions have been implemented, often with sparse or low-quality research. The National Standard of Canada for Psychological Health and Safety in the Workplace (the Standard) is a comprehensive framework aimed at promoting mental health and preventing psychological harm in Canadian workplaces. This longitudinal multiple-cohort implementation science project describes mental health strategies implemented and associated organizational outcomes across five PSP organizations implementing change within the standard framework. Data were collected at two levels over a three-year span from the five public safety organizations that identified priority areas for improvement within the Standard based on local data and consultations. The organization selected and implemented a range of proactive mental health interventions, including resiliency training. Individual pre-post surveys assessed a variety of mental health disorders and work-related items. Annual organizational data included sick leave hours and extended health benefits for psychological services. Survey responses were aggregated at the organizational level. Rank-based correlation analyses (Kendall’s tau) described associations among occupational stress, work engagement, stigma, and organizational indicators. Organizations demonstrated multiple indicators of progress in meeting the Standard. Post-mental health symptom scores were positively correlated with extended health costs. Higher organizational stress scores were associated with higher extended health costs (psychological) (τ = 1.0 at pre-intervention; 0.67 post-intervention). Positive changes in organizational stress scores and higher engagement scores over the implementation process were both associated with lower average extended health costs (τ = 1.0/−1.0 respectively). Resilience scores were inversely related to health costs (τ = −0.67), consistent with the protective role of resilience. The Standard can serve as a framework for improving workplace health and safety when integrated with multi-modal action plans and structured resilience programs.

## 1. Introduction

Public safety organizations increasingly use workplace mental health interventions to address their employees’ well-being [1], yet longitudinal evidence supporting these interventions remains limited. Further, interventions tend to focus on the diagnosis and treatment of illness and injury once symptoms are present, downstream from the actual onset. Less attention is paid to mitigation strategies found on building skills prior to the onset of injury [1,2]. Although “preventing” post-traumatic stress injuries is impossible, learning a suite of mitigation strategies may build resilience to illness and injury, lessening their impacts [3].

Through the course of their work, Public Safety Professionals (PSP) are regularly exposed to potentially psychologically traumatic events (PPTEs; i.e., direct or indirect experiences of actual or threatened death, serious injury, or sexual violence [4]) which may predispose these professionals to increased incidents of post-traumatic stress injuries (PTSIs) [5]. Further, death by suicide among a group of police, firefighters, paramedics, and correctional workers appears to occur at a higher rate when compared to the general population [6]. In response to emerging information, federal, provincial, and territorial governments in Canada have been developing new strategies, legislation, and a national framework to better support PSP with PTSIs [7]. Subsequently, there has been a proliferation of mental wellness programs for PSP; however, an evidence base of research detailing program effectiveness and consistency of implementation is necessary [8]. In a systematic review and meta-analysis, Di Nota et al. [1] found multi-modal psycho-education programming to produce modest, time-limited reductions in symptoms of major depressive disorder, burnout, stress, post-traumatic stress disorder (PTSD), and generalized anxiety disorder, while promoting general psychological health, adaptive coping, and resilience, lasting up to 18 months.

Following the publication of data suggesting PSP [5] show high prevalence of positive screens for mental health disorders, in comparison to the general Canadian population, and in response to several Ontario police officer deaths by suicide over a short span of time, the Ontario Coroner’s Office [9] expert panel report suggested a whole-of system approach and joint ownership and collaborative action to support to positive outcomes in police member mental health. These data and recommendations provided the impetus for an ecological model of resilience, informed by Resilience Theory [3] (Van Breda, 2018) and Ecological Theory [10], and consideration of the role of the National Standard of Canada for Psychological Health and Safety in the Workplace (hereafter called the Standard) to guide collaborative action.

### 1.1. Resilience

Resilience promotes recovery, or a return to a previous state of well-being following exposure to a stressor [11]. Resilience Theory explores mediating processes that encourage engagement in adaptive behaviors when facing stressors, including PPTEs. Van Breda [3] emphasized resilience as a process that leads to an outcome (being resilient), and the target of resilience research should then explore the mediating process enabling better-than-expected outcomes. Here, resilience was often individualized without consideration for the broader context. However, Hartling [12] extended interpretation by finding resilience processes extend beyond individuals to networks of social relationships.

Conceptualizations of resilience, focused on risk and protective factors [13], were redefined as emerging through ongoing transactions within networks of individual, familial, and community systems [14]. The shift from models emphasizing individual capacities to multi-systemic, contextualized conceptualizations [15] raised awareness that individual resilience interacts with external supports that ameliorate risk. Thus, contemporary understanding of resilience requires recognition of the mediating roles of broader social systems to be effective.

Bronfenbrenner’s Ecological Systems theory framed a public health response as representing the convergence of biological, psychological, and social scientific considerations, recognizing the importance of interactions between the individual and their environment [16]. Stokols [17] suggested that such a model enables simultaneous emphasis on individual and contextual systems while acknowledging the interdependent relations between the two. In response, the proposed ecological model of resilience examines how interrelated systems (i.e., their self, families, and workplaces) simultaneously impact individual resilience.

#### An Ecological Model of Resilience

Definitions of resilience interpret the concept as “rebound from,” “adapting or adjusting to” adversity, or other terminology indicative of an immunity to negative outcomes after adversity, yet there is “no gold standard measure” of resilience as a singular construct [18]. Historically, resilience was described as an individual’s “resistance to psychosocial risk experiences” [19] (p. 119), conflating resiliency with resistance and suggesting an invulnerability or innate capacity for successful adaptation despite compromising circumstances [20]. Resilience and resistance are increasingly argued as distinct constructs—resilience being the capacity to rebound and resistance being the capacity to not be impacted in the first place (i.e., the “invulnerability”). In developmental psychology, resilience focuses on the hardiness of individuals who cope well with extreme stress [18,19] or adversity [13]. Here, resilient individuals were thought to possess “steeling effects,” or innate, self-righting traits [18] that buffer external stresses, which is currently more aligned with interpretations of resistance, rather than resilience. Resilience, comparatively, is a fluid construct related to an individual’s recovery, or a return to a previous state of well-being following exposure to a stressor [11]—not an immunity. Resilience extends beyond individual personality traits and involves behaviors, thoughts and actions that can be learned and developed.

Psychological resilience, then, is understood as influenced by subjective perceptions of personal support and acceptance, with appropriately tailored help available as required [21]. Given that any organizational environment exercises a powerful influence on reactions to PPTE [22], workplace PTSI prevention pillars that include psychological and social support, organizational culture, clear leadership and expectations, workload management, policies, educational initiatives, return-to-work accommodations, and many others are deemed essential [23,24]. The National Standard of Canada on Psychological Health and Safety in the Workplace includes mental wellness organizational standards, guidelines, and evaluation tools [25], which have been adapted for organizations employing paramedics [26]. To support resilience, family members play a central role as they are likely the first to notice the signs and symptoms of PTSIs and can provide the support and encouragement needed to link individuals to help and resources [5]. In conceptualizing a resilience ecosystem that supports PSP psychological health and well-being by recognizing the roles of personal, family and workplace contributions, we show how an ecological system can empower PSP while providing fulsome support, and in consequence encourage help-seeking required.

**Individual Resilience:** Relating resilience to recovery, or a return to a previous state of well-being following exposure to a stressor [11], is the practice of rebounding from adversity with new skills and strengths that, if not for adversity, would not have developed [11]. Thus, teaching PSP skills to grow from PTSI exposure can help mitigate future risks associated with working in an adverse and unpredictable environment. Programs targeting resilience in PSP, within this interpretation of resilience, often provide training to an individual to improve mindfulness, stress management, coping skills, and emotion regulation, using psychoeducation or cognitive behavioral therapy [1]. Indeed, of the resilience promotion programs that included skill-building, most produced modest, time-limited improvements in several PTSI- and PTSD-related measures. Specifically, 24 of 35 studies reviewed by Di Nota et al. [1] were of moderate to low quality, while 14 had a high risk of bias.

Overall, there is limited evidence-informing resilience programming for PSP [21] and general adult populations [27]. Recent results reveal potential benefits of resilience training for mental health and well-being, particularly through cognitive behavioral therapy and mindfulness-based interventions, or a mix of both [28]. To date, however, psychoeducation interventions have had mixed reviews, beyond criticism for responsibilizing the individual for their “recovery” rather than recognizing the ecosystem around the individual and its role. Having said that, through a successful RCT study with cohorts of primary care paramedic students using recognized psychometric resilience measures, Anderson, Vaughan, and Mills [29] demonstrated favorable individual improvements in resilience following online training in hopes to better prepare students to move into active deployment.

**Family Resilience:** Given that family members are likely to notice the symptoms of PTSIs in their PSP family member, they are invaluable to encouraging PSP to seek help and access available resources [30], although emergent models of family resilience reveal that the path a family follows to adapt to stress and trauma varies widely [31]. Integral to resilient families finding and following their “path” is intra-familial processes, specifically maintaining shared beliefs, family organization, and communication [32,33]. Shared beliefs can be introduced or reintroduced to the family system through religious, spiritual practice, artistic expression, or through connection with family rituals or the natural environment, always serving to help family members make sense of stress and adversity. Walsh [33] contends that agency and self-determination are mobilized through shared belief systems and are instrumental in efforts to reinterpret adversity as a challenge to be mastered. Family organization enables families to manage adversity’s implications, particularly when clear and consistent, the organizational patterns reassure family members of the continuous structure, dependability, and strength of the family system [33]. Communication processes, when messages are unambiguous, empathetic, and openly shared, encourage inclusion, collaborative problem-solving, and conflict resolution, are also valuable for resilient families.

Paralleling conceptualizations of individual resilience, models of family resilience have evolved to emphasize context, developmental level, and the interactive combination of risk and protective factors [31]. Family resilience models have involved through two previous “waves” with the third in progress [34]. Wave 1 family resilience models were based on family stress and coping theory [35], depicting resilience as an outcome resulting from the “steeling effects” [36] possessed by families able to withstand normative and non- normative stresses. Wave 2 models emphasized resilience as a process [32] resulting from the interplay of individual assets and the social contexts in which they developed [37]. Here, for families experiencing adversity, resilience processes enable them to recover or “bounce back” by developing “resistance to psychosocial risk experiences” [36] or to reconfigure through forward motions [11] as new capacities are developed and adversity overcome. The current Wave 3 model of family resilience places increased emphasis on cascades and trajectories of risk, promotive and protective factors, family vulnerabilities, and adaptation over time [34]. Because resilience science is valued, consolidation and testing of proactive secondary programs and policies focusing on family resilience are priorities in Wave 3 [34].

**Workplace Resilience:** Research demonstrates how a workplace contributes to an individual’s stress [38,39,40,41]. Particularly, organizational environments influence how traumatic stress reactions are experienced [22]. Further, workplace stressors are qualitatively found to be more challenging and harmful than operational stressors [38]. Thus, workplace PTSI prevention pillars are growing in normalization and oft include psychological and social support, organizational culture, clear leadership and expectations, workload management, policies, educational initiatives, return-to-work accommodations, among others [23,24]. Ip et al. [41], for example, examined workplace psychological safety, finding organizations investing in leadership development, supportive workplace climates, and balanced hierarchical structures with inclusive decision-making can help ensure workplace environments foster increased performance, well-being, and organizational resilience.

Like family resilience, maintaining shared beliefs, a culture of organizational support, a fair and equitable distribution of work, and transparent communication play a role in organization resilience [23]. For PSP, research supports unit cohesion as a fundamental cornerstone to an organization’s resilience [42]. For example, the characteristics of the firefighter community, like living together during their shifts where they can interact as a group around a table after calls and the conditions under which firefighters work, provide a foundation for workplace resilience.

Conceptualizations of workplace resilience, conceptualizing the workplace as a community, emphasize the attributes a community acquires when faced with expected and unexpected opportunities and challenges [42]. Workplace resilience develops from collective competence and shared responsibility, the former refers to the actions of community members taken address common needs or to confront situations that threaten their well-being [42]. The latter, shared responsibility, develops when community members commit to demonstrating concern for the welfare of other community members [42], a principle referred to as “unit cohesion” within military institutions [43].

Collective competence and shared responsibility function interdependently to build “social capital,” referring to a collection of resources developed through reciprocal relationships between informal and formal networks [44]. Mobilizing community capacities to achieve goals and develop and main resilience requires social capital and a shared vision.

### 1.2. The Standard

**Figure 1:**
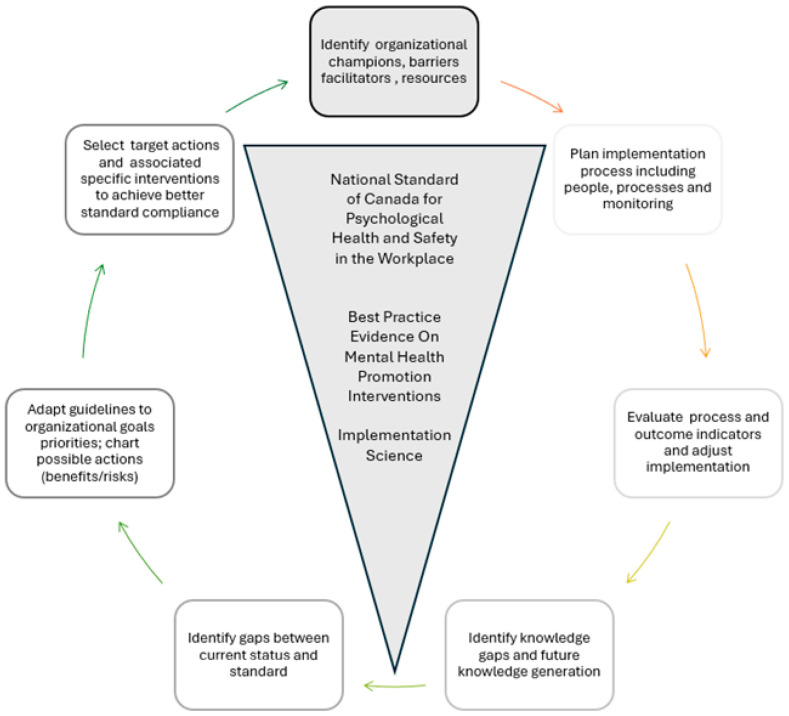
An adapted Knowledge to Action Cycle (adapted from [45]).

The National Standard of Canada for Psychological Health and Safety in the Workplace [25] is a set of voluntary guidelines, tools, and resources designed to help organizations promote mental health and prevent psychological harm at work. It was first launched in January 2013 and reaffirmed in 2022 and expected to be updated in 2026. the Standard provides a framework for establishing a Psychological Health and Safety Management System focused on three strategic pillars: prevention of harm—identifying and mitigating workplace hazards that cause psychological injury; promotion of health: actively supporting mental well-being for all employees; and resolution of incidents: creating clear processes for reporting and addressing psychological concerns. The Standard identifies 13 key organizational factors that impact employee mental health, including factors like organizational culture, workload management, and psychological protection. These factors are used to assess the current environment, identify hazards, and implement improvements to support a psychologically safe workplace. While not specific to PSP the Standard provides a structure and process that can be used by organizations for quality improvement which by definition requires identifying internal priorities for action using the standard as a framework for a gap or needs analysis, tailoring best practice evidence into local targets and implementation plans, identifying barriers/facilitators that have to be addressed, proceeding with implementation and evaluation of impact. The Knowledge to Action Cycle [45] is useful for guiding implementation in the PSP community, when combined with more specific evidence about best practice (see Figure 1 for adapted KTA cycle [46].

The Standard is intended to help organizations “recognize psychological health as a part of an ongoing process of continual improvement” [25] (p. 8). The standard was then adapted to specifically provide a systematic approach to identifying, assessing, mitigating, and reducing psychological harms that are unique within paramedic service [26] (and more in line with other public safety organizations).

Both the Australian and EU standards also recognize violent or traumatic events as a risk (and include remote or isolated work in their list) for paramedics. While recognizing barriers for implementation of the Standard (e.g., leadership support, data access and evaluation, definition of “excessive stress”), the Mental Health Commission of Canada [47] reviewed the voluntary adoption and implementation of the standard in 40 organizations, demonstrating that the standard can be an effective tool to help improve the psychological health outcomes for their employees. Implementation of the standard “helps an organization to identify and mitigate hazards that can contribute to psychological harm to the worker” [25] (p. 9). Thus, having a committee responsible for the implementation of the standard creates a venue for ongoing discussions related to employee mental health and well-being, compliance, and risk mitigation strategies, all intended to reduce mental illness was a key foundation for this study’s success.

### 1.3. Purpose

The aim of this research project is to promote resilience in fire and police organizations to reduce the risk of occupational stress injuries and support long-term psychological well-being among public safety employees. While many short-term pre-post intervention studies have examined psychoeducational programming to increase resilience, mental health and well-being, few longitudinal studies exist. Using an integrated knowledge translation (iKT) case series design involving the co-creation of research questions, methodologies, data collection, analysis and interpretation with full participation of knowledge users throughout the entire research process [48], this research aimed to: (1) introduce a new conceptualization of resilience to public safety professions; (2) co-create and deliver psychoeducational content directed at each pillar of the ecosystem which supports PSP resilience (personal, family, and workplace resilience factors); (3) determine the impacts of sustained efforts supporting a psychologically safe workplace through the implementation of the Psychological Health and Safety in the Workplace (Z1003) standard on organizational culture and mental health of employees by providing a systematic approach to identifying, assessing, mitigating, and reducing psychological harms that are unique within police and fire services; and (4) demonstrate the long-term benefits of implementing training and planned departmental efforts in reducing psychological injury costs and absenteeism due to illness.

## 2. Materials and Methods

Five public safety organizations participated in this project, including three municipal police departments and two fire rescue services located from the Lower Mainland of British Columbia. These organizations varied in size, operational context, and existing capacity to support psychological health initiatives. Despite these differences, all five agencies shared a common interest in advancing workplace mental health and resilience through structured, evidence-informed programming.

**Figure 2:**
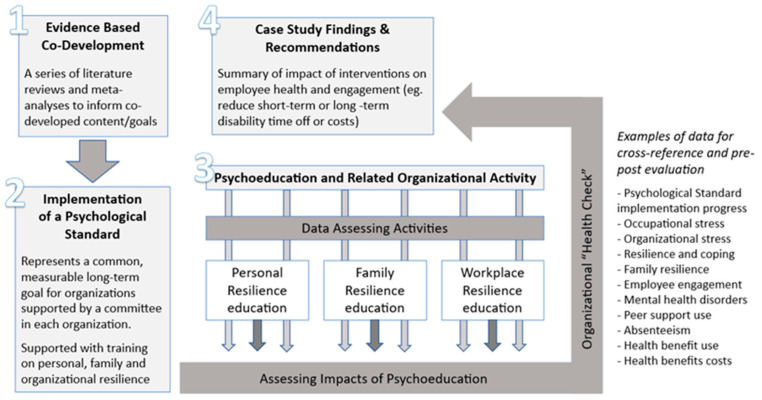
An overview of the research process which was common for all organizations.

Each organization engaged in a voluntary, internally driven process, supported by external facilitation and resources provided through the project team. While the overarching goal was consistent—to foster psychologically safer workplaces and support resilience at multiple levels—each department set its own implementation priorities based on local needs, capacity, and internal assessments. Supports were provided by the project team to help organizations develop structured psychological health strategic plans with strategies, tactics and key performance indicators to help guide efforts and measure success (refer to Figure 2).

Over a three-year period, the project co-developed and implemented initiatives in a way broadly consistent with the KTA cycle although process is typically iterative and varied across organizations with a shared goal to enhance psychological health and safety in the workplace consistent with the Standard. These initiatives were grounded in three key components:

- Examining and addressing gaps in organizational policies and procedures related to psychological health and safety;
- Supporting the familiarization and implementation of the Canadian Standard for Psychological Health and Safety in the Workplace;
- Developing and delivering psychoeducational training tailored to public safety personnel, civilians, and leadership, structured around the three pillars of resilience: individual, family, and workplace.

To assess the implementation and impact of these interventions, data collection focused on three complementary streams:

- Psychological health and well-being surveys: Administered at baseline and approximately two years post-training, these surveys assessed changes in employee well-being and perceived organizational culture.
- Psychoeducational training courses: Three online training modules—Individual Resilience (Course 1), Family Resilience (Course 2), and Organizational Resilience (Course 3)—were delivered across all participating organizations. Standardized psychological instruments were used to evaluate course-specific outcomes before and after training.
- Organizational records: Human resources and operational data were collected to explore organizational-level trends in absenteeism, benefits usage, and other indicators relevant to workplace stress and wellness.
- Psychological standard implementation gap analysis: The Guarding Minds at Work survey was used to look at the 13 psychological factors present in the National Standard. This survey includes 28 recommended practice areas (sections) comprising 68 statements about common work experiences covering work responsibilities, relationships, and leadership that can be directly mapped back to the psychological factors present in the National Standard. Each question was answered with not implemented (0), partially implemented (1) and fully implemented (2). For each recommended section, the score was calculated as a percentage of the total statement scores relative to the maximum possible score if all items were fully implemented.

### 2.1. Psychological Health and Well-Being Survey

To evaluate the broader impact of implementing the Canadian Standard for Psychological Health and Safety in the Workplace, as well as three targeted training interventions, a comprehensive psychological health and well-being survey (PHW survey) was administered at two time points: prior to implementation (baseline) and two years post-intervention. This component of the project aimed to assess organizational-level changes in employee mental health, resilience, workplace culture, and psychological safety.

The survey combined standardized clinical screening tools with occupational mental health instruments, providing a multidimensional view of individual well-being, perceived workplace conditions, and organizational stressors. The selected instruments were validated for use in occupational and public safety populations and covered domains such as PTSD, and symptoms of anxiety, depression, social phobia, alcohol use, as well as work engagement, and resilience. Table 1 summarizes the instruments used in the PHW surveys.

This organizational-level survey served two primary purposes. To establish a baseline profile of psychological health, resilience, and workplace attitudes across the participating public safety organizations; and to assess changes over time in response to the introduction of the Standard and accompanying training and other interventions.

All data were collected anonymously and analyzed at the aggregate level. The integration of this data with other project components supports a comprehensive ecological view of resilience in public safety workplaces and provides critical insight into the systemic impact of psychological health interventions.

### 2.2. Organizational Records

To complement the survey and training data, organizational-level records were compiled from each participating fire and police agency to provide additional context for understanding trends on staffing levels, operational pressure, financial investment in employee health, and key indicators of organizational performance. These data were aggregated from publicly available sources like annual reports and the human resources department. Where applicable, additional internal records were shared by participating agencies at their discretion.

These records include information on staffing levels, operational activity, health and benefits utilization, and critical incident supports, offering a multi-year snapshot of workplace conditions that may influence or reflect mental health outcomes in public safety settings. Collecting these metrics provides insight into the broader organizational environment in which resilience programming was introduced. A summary of organizational records is shown in Table 2.

### 2.3. Data Cleaning of PHW Responses

The survey employed a forced-choice format to prevent missing items within scales. Incomplete responses were handled via available-case analysis: respondents who exited the survey early were excluded only from the specific analyses involving the missing instruments, while their completed data for other validated scales were retained.

To improve data integrity, a multi-step cleaning process was applied to the PHW survey responses. First, the fastest 2% of responses were removed based on average response time per item (sec/item), a well-established index for detecting careless or insufficient effort responding [56,57]. This distributional approach yielded a threshold of approximately 3.5 s per item for both surveys (T1 and T2), above the conventional 2 s/item minimum benchmark [57] to accommodate the length of our survey questions. Next, additional responses were excluded if they met the top 5% of fastest completion times and any one of the following criteria, following the multi-indicator approach recommended by [56]:

- More than 80% of symptom items scored as zero;
- Two or more low inter-scale correlations between PHQ-9, GAD-7, and PCL-5 (defined as correlation outliers using the 1.5 × IQR rule);
- Outlier scores on more than two psychological scales (also based on the 1.5 × IQR rule).

### 2.4. Potential Associations Among Survey Responses and Organizational Outcomes

To support evidence-informed decision-making on workplace mental health investment, this section explores potential associations between employee self-reported survey responses and organizational-level indicators, such as sick leave and psychological health-related benefit costs. The aim is to understand how proactive mental health strategies may relate to organizational well-being and offer potential returns in the form of reduced costs and improved performance.

### 2.5. Data Sources and Aggregation

PHW Surveys included measures of occupational stress, work engagement, stigma and standardized screening tools for mental health disorders. At the organizational level, a range of administrative indicators were collected annually; however, not all metrics were consistently available across all participating agencies. To support comparability and align with priorities at the management level—particularly around return on investment (ROI)—we focused on two key indicators: sick leave hours, as a proxy for absenteeism, and extended health benefit costs related specifically to psychological services.

To enable cross-level analysis, survey responses were aggregated at the organizational level to generate average scores on key mental health and occupational constructs. These were then compared to organizational indicators to examine possible associations and trends over time.

### 2.6. Analytic Approach: Rank-Based Correlations

Given the small number of participating organizations and the non-normal distribution of certain metrics, non-parametric rank-based correlation analyses (Kendall’s tau) were used. This method evaluates the strength and direction of association between ranked variables and is more robust to outliers and small sample sizes than parametric alternatives. This approach provides a conservative estimate of monotonic relationships between variables. While this approach allows for the identification of strong directional trends, we report these values as exploratory measures of association. Correlations were calculated at:

- T1 (pre-intervention);
- T2 (post-intervention);
- T2 − T1 change scores (i.e., shifts over the intervention period).

For T1 and T2, organizational indicators (i.e., sick leave hours and extended health costs) reflect data from the corresponding individual year. However, for T2 − T1 change scores, we used the multi-year average of organizational indicators over the intervention period (i.e., 2022–2024) to better capture trends and smooth out year-to-year variability. This approach supports a more stable comparison with aggregated survey score changes and facilitates meaningful interpretation of associations.

## 3. Results

At T1 (Fall 2022), a total of 372 individuals participated in the PHW survey battery, including 124 from police agencies and 248 from fire departments. At T2 (Winter 2025), 207 individuals participated in the follow-up survey, with 134 from police agencies and 73 from fire services. Following data cleaning, 14 responses were excluded from the T1 dataset (*n* = 386) and 6 from the T2 dataset (*n* = 213). This procedure helped ensure that analyses were based on attentive, valid self-reports. Notably, one participating fire agency did not take part in T2, which contributed to the reduced fire sample size at follow-up.

Two specific instruments—the Resilience Scale for Adults (RSA) and the Team Psychological Safety Scale (TPS)—were not included in the original T1 survey. Instead, data from pre-course survey responses were used as baseline comparison points for these measures in Spring 2023 and Fall 2024, respectively.

### 3.1. PHW Survey Results

The comparison of survey results includes survey responses collected in 2022 (T1, *n* = 372) and 2024 (T2, *n* = 207), with subgroup results for police and fire agencies. Across the full sample, three of the five participating organizations demonstrated improvements in the majority of indicators (more than 8 out of 11 measures). At the overall level, statistically significant improvements were observed in anxiety, alcohol use, and team psychological safety (TPS).

Organization-level analyses identified targeted areas of significance: operational stress improved in two organizations, while anxiety, alcohol use, and work engagement showed significant improvements in single organizations, respectively. Most notably, aggregate-level analyses (Table 3) revealed statistically significant improvements in three critical domains: anxiety symptoms, alcohol consumption, and team psychological safety.

However, deteriorations were observed in individual resilience scores. This pattern is consistent with the decay of acquired skills often observed in educational interventions, where unreinforced gains diminish over time [61]. Notably, the individual resilience training module was administered in 2022; the temporal lag between intervention and the T2 2005 survey administration may have limited the detectable impact at follow-up due to skill decay. An increase in social interaction phobia was also documented. Two contextual mechanisms are offered as potential factors due to the time frame of this study: first, post-COVID re-entry effects, characterized by elevated anxiety as in-person demands resumed following initial reintegration; and second, heightened workplace visibility where frontline personnel face increased scrutiny via public recording and social media exposure, potentially exacerbating interactional self-consciousness.

Collectively, these findings present a mixed but predominantly positive profile, highlighting significant gains in key domains alongside context-specific areas requiring sustained reinforcement.

At the group level, the police sample showed improvements in several domains, particularly operational stress, anxiety, and TPS. The fire services group exhibited more pronounced improvements across most indicators, including significant reductions in operational stress and anxiety. However, results also revealed substantial variation across individual agencies, with some showing greater improvement on PHW average scores than others. These differences likely reflect a combination of factors, including organizational readiness, implementation strategies, and local context, underscoring the importance of tailoring resilience-building efforts to each agency’s unique environment.

“Negative comments from the public” was among the top operational stressors across police services. In fire services, “shift work” and “not enough time with family and friends” ranked among the top stressors.

### 3.2. Training Outcomes

Table 4 shows the pre-post survey mean score comparison for the resilience training courses. The Resilience Scale for Adults (RSA) measures protective resilience factors, with higher scores indicating greater resilience capacity. The Coping Strategies Inventory—Short Form (CSI-SF) assesses coping style across two subscales: engagement strategies (CSI.E), which reflect adaptive approaches that directly address stressors, and disengagement strategies (CSI.D), which offer short-term relief but may lead to longer-term challenges.

The family resilience training module was designed to promote shared understanding among PSPs and their families, particularly in relation to the challenges of shift work, trauma exposure, and occupational stress. The Walsh Family Resilience Questionnaire (WFRQ) measures core dimensions of family resilience, including belief systems (Belief), organizational patterns (Org.), and communication (Comm.). Data from the one-month follow-up (T2) were excluded from this analysis due to insufficient participation (*n* = 2).

As shown in Table 4, results indicate a short-term impact on the average scores. Specifically, mean values for individual resilience and engagement coping strategies (CSI.E) increased one month post-course (T2), alongside a reduction in average disengagement coping (CSI.D). However, these aggregate improvements were not sustained at the four-month follow-up (T3). This pattern suggests that while the individual resilience training prompts immediate positive shifts, these gains may diminish without continued reinforcement (see [61]). In contrast, participants’ perceptions of family adaptability, structure, and communication showed sustained increases from baseline (T1) to the four-month follow-up (T3), implying a more durable impact from the family resilience training. However, the limited sample size at follow-up necessitates caution when drawing definitive conclusions from these trends.

### 3.3. Correlation Analysis

Table 5 summarizes strong correlations (Kendall’s tau > 0.6 or <−0.6) observed between aggregated survey measures and organizational indicators. While five organizations participated in the study, extended health cost data were unavailable for one organization; consequently, results involving this indicator were calculated based on the remaining four organizations. Organizational stress consistently showed strong positive associations with extended health costs at both time points and across change scores (τ = 0.67), suggesting that higher perceived stress within the organization may drive greater use of psychological health benefits. Work engagement showed a strong inverse association with extended health costs over time (τ = −0.67), reinforcing the idea that improvements in engagement may reduce healthcare expenditures. Mental health symptom scales (e.g., PTSD, anxiety, depression) at T2 and the change were consistently observed to be positively correlated with extended health costs, aligning with expectations. Resilience scores were inversely related to health costs at both T2 and T2 − T1 (τ = −0.67), consistent with the protective role of resilience. However, the negative correlation between depression and costs at T1 (τ = −0.67) and the positive correlation between team psychological safety and costs at T2 and T2 − T1 (τ = 0.67) were unexpected and may reflect differences in awareness, access, or reporting patterns.

**Figure 3:**
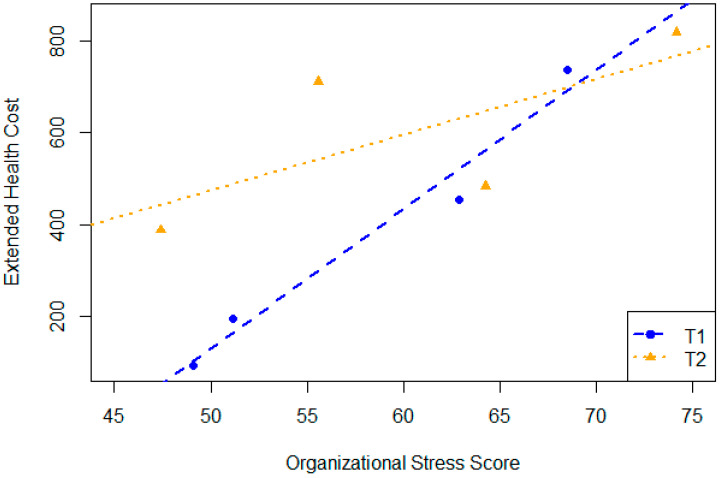
Relationship between extended health costs and stress levels across organizations.

The positive directional trend across all organizations is seen in Figure 3. The plot shows that as the organizational stress score increases, extended health costs trend upward for both T1 and T2 groups.

## 4. Discussion

The current study introduces a novel, more holistic ecological approach focused on examining how different PSP organizations implement standards using individualized tailoring as well as engagement in core resilience training intended to address occupational stress at the individual, familial, and workplace levels. The model was developed following a series of structured reviews examining mental health disorders, mitigation strategies, coping and resilience in PSP, e.g., [1,2,21]. The ecological model shifts from models emphasizing individual capacities to multi-systemic, contextualized conceptualizations, which raises awareness that some individuals may not be resilient, not because they lack agency, but because they may be disconnected from the protective factors and supports that ameliorate risk [13,15].

The presented findings underscore the potential value of implementing sustained, tailored mental health strategies that include a broader conceptualization of resilience that recognizes factors beyond the individual (including family and workplace), and those guided by recognized frameworks such as the Canadian Standard for Psychological Health and Safety in the Workplace as demonstrate by the improvements in the majority of indicators (more than 8 out of 11 measures) in three of the five participating organizations. By supporting structured approaches to promoting organizational wellness, such standards help account for the complex interplay of individual, cultural, and systemic factors across diverse public safety settings.

A National Standard for Psychological Health and Safety in the Workplace can serve as a framework for improving workplace health and safety when integrated with multi-modal action plans and structured resilience programs implemented by engaging organizations in a quality improvement process that jointly improves organizational and health outcomes. The extant literature suggests unidimensional time-limited solutions are unlikely to produce the broad improvements hoped for by PSP, their leaders, and their families [1,21]. The challenges experienced by PSP are multidimensional and prolonged, from training to beyond retirement [8], which means effective solutions will need to be multidimensional and longitudinal. Any effective solutions will need to engage and coordinate individuals, their leaders, their organizations, and their families, as well as several levels of government [8]. Multidimensional interventions, such as those built for the Royal Canadian Mounted Police Longitudinal PTSD Study [62], PSPNET [63], and PSPNET Families [64], can be important tangible deliverables. The next steps will involve strategic advances that deliver tangible, iteratively evaluated and improved interactive solutions. The current model can help inform those steps as part of reaching the aspirations described in the National Standard.

The key to long-term success is the scientific approach. Integrated Knowledge Transfer research addresses knowledge users’ real-world problems through the co-creation of knowledge and its application in addressing the issue at hand. Graham et al. [45] consider it to be an “implementation science in cases where generalizable knowledge has been created about how to facilitate research use (p. 21).” Integrated Knowledge Transfer required a collaborative approach to the research design, outcome measures, data collection, implementation and evaluation, which should include the generation of appropriate messaging of results, application of findings, and dissemination to the knowledge users’ organization and peers. Knowledge users and researchers were equal partners in a research process designed to be action-oriented and solution-focused, increasing the likelihood that results will be broadly applicable to the knowledge user and their work context [45]. Answering specific questions posed by the knowledge users (resulting in systematic literature reviews) and the development of psychoeducation material through the use of a knowledge-to-action cycle provided a clear path for input from all parties and active participation.

Psychoeducational material was developed in response to departmental concerns and the broader ecological model of resilience. Mental health injuries cost the Canadian economy approximately $15 to 33 billion every year in disability costs alone and cause the affected individual’s personal distress [65]. Further, delay in seeking treatment due at least in part to the stigma surrounding mental health can increase the cost burden, as the individual’s condition worsens without treatment [65]. Preventative strategies, such as education about mental health or “psychoeducation,” can reduce stigma and may be a solution to the large-scale costs of mental health disorders [1,65]. Researchers have demonstrated how psycho-educational programs in the workplace have a nine-to-one return on investment [66]. Included here in the return is how psychoeducation may also influence workplace culture by reducing stigma and increasing mental health literacy skills, and can help individuals learn prevention strategies, recognize developing disorders, become aware of treatment and support options, and have the skills to provide mental health first aid to those in need [65]. Implementing psycho-education programs developed for and with PSP through a knowledge-to-action cycle provided direct benefit to employees and reduced the strain on the mental health care system, yielding substantial economic and health benefits [67].

The present results suggest psychological stress within the organization is a key driver of health benefit utilization, as higher levels of perceived stress were strongly associated with increased extended health costs. Conversely, higher levels of work engagement and resilience were associated with lower extended health costs, suggesting potential pathways toward enhanced performance and reduced psychological service expenditures. However, no strong associations were observed between any survey indicators and sick leave hours, which may reflect differences in reporting practices, types of leave captured, or limitations in the available data (for example, firefighters often trade shifts rather than take sick days). Additionally, some unexpected associations emerged, such as a negative correlation between symptoms of depression and benefit costs at baseline (potentially influenced by initial stigma associated with utilizing benefits), and a positive correlation between team psychological safety and benefit costs at T2 (which may indicate a greater willingness to use benefits following periods of mental health training), indicating the need for deeper exploration of various factors.

### Limitations

Importantly, these analyses are descriptive and exploratory and are constrained by the small number of participating organizations and limited follow-up data points available for analysis. Specifically, in a small-N context, rank-based coefficients can yield perfect values (e.g., τ = 1.0) if the organizational rankings align perfectly across indicators. While these results highlight strong directional trends within the studied group, they lack the statistical power for broad generalization. Therefore, these findings should be interpreted as indicative of potential associations that warrant further investigation in larger-scale studies.

Data collection using the PHW survey could include self-selection bias and biased attrition. Further, this study was delayed due to COVID-19; therefore, the timing may have some impact on survey results. Our previous work found the COVID-19 pandemic to be a risk factor for increased mental health symptom reporting in PSP [68]. Timing and heightened concerns post-COVID-19 may play a role in SIPS and depression scores (which may well have been increased during COVID-19), contributing to unexpected trends.

Finally, in the PSP community, it is common for employees to manage health issues informally, which masks administrative sick leave data and may limit the association between mental health and the utilization of sick leave. In our cohorts, fire services personnel reported a ‘culture of presenteeism’ or ‘shift-swapping’ to cover shifts during short-term illness that introduces a legitimate confounding factor in the analysis.

## 5. Conclusions

A National Standard for Psychological Health and Safety in the Workplace can serve as a framework for improving workplace health and safety when integrated with multi-modal action plans and structured resilience programs implemented by engaging organizations in a quality improvement process that jointly improves organizational and health outcomes. It also highlights the limitations of one-time training due to skill decay [61]. Skill maintenance is required following an investment in training. Organizations should consider modifying their training programs to ensure sustained effects by introducing potential ‘booster’ sessions, continuous post-training mentorship or something as simple as email “touch points” for the purpose of having PSP reflect on their training. Implementation of the National Standard may provide structures through which sustained efforts are supported.

While this type of research is difficult to “control,” time-consuming and subject to data collection gaps, it is highly generalizable to the real-world context and does provide early insight into how implementing psychological health standards and resilience-based training might relate to broader organizational outcomes. The findings support ongoing evaluation and adaptive implementation as a key element in implementing standards in complex occupational contexts.

## Figures and Tables

**Table 1:** Instruments used in PHW surveys.

Instrument	Construct Measured	Score Range	Interpretation
Opening Minds Scale for Workplace Attitudes (OMS-WA)	Attitudes toward stigma in the workplace [46]	11–55	Higher scores indicate higher attitudes toward stigma in the workplace
Operational Stress Questionnaire (PSQ-Op)	Operational stress (e.g., shift work) [39,47]	20–140	Higher scores reflect greater operational stress
Organizational Stress Questionnaire (PSQ-Org)	Organizational stress (e.g., leadership) [39,47]	20–140	Higher scores reflect greater organizational stress
Utrecht Work Engagement Scale (UWES-9)	Work Engagement [48]	0–54	Higher scores indicate increased work engagement
PTSD Checklist for DSM-5 (PCL-5)	Post-traumatic stress disorder symptoms [49]	0–80	Higher scores reflect greater PTSD symptom severity
Generalized Anxiety Disorder Scale (GAD-7)	Anxiety symptoms [50]	0–21	Higher scores indicate higher levels of anxiety
Patient Health Questionnaire (PHQ-9)	Depression symptoms [51]	0–27	Higher scores indicate more severe depressive symptoms
Social Interaction Phobia Scale (SIP-S)	Social anxiety [52]	0–56	Higher scores indicate increased fear of social interaction
Alcohol Use Disorders Identification Test (AUDIT)	Risk of alcohol misuse or dependence [53]	0–40	Higher scores indicate increased risk of alcohol use problems
Resilience Scale for Adults (RSA)	Resilience score [54]	33–165	Higher scores indicate higher levels of protective resilience factors
Team Psychological Safety (TPS)	Team Safety [55]	7–49	Higher scores indicate shared belief that the team is a safe environment for interpersonal risk-taking

**Table 2:** Summary of organizational records.

Data Category	Specific Metrics	Notes
Geographic context	Population served (annual); jurisdiction area (sq km)	Reported annually for both police and fire
Staffing	Authorized and actual staff numbers by role	Police: sworn, civilian, executive; fire: suppression, prevention/education, training, exempt
Human resources records	Overtime hours; sick leave hours; long-term leave hours	Reported annually by staff category
WorkSafe BC claims	Number of claims, type of claim (e.g., mental disorder vs. other), claim cost, days lost, return-to-work	Available for police only; not consistently reported for fire departments
Extended health benefits	Annual cost of health services by category	Includes prescription drugs, paramedical services, and psychological care; reported for both police and fire
Post-incident support	Number of peer support sessions, debriefings, and check-ins (including deployment-related)	Applicable only to police
Critical/incident reports	SBOR reports, assault PO files, PCMVI	Police-specific indicators of frontline stress exposure
Oversight activity	External investigations (e.g., IIO, OPCC)	Applicable only to police
Calls for service	Annual number of calls (total and by type)	Police: general occurrence reports, CAD cleared reports; fire: medical and non-medical calls

**Table 3:** Mean scores, standard deviation, and Cohen’s d of PHW survey responses (T1 vs. T2).

Mean (SD)	T1 *n* = 372	T2 *n* = 207	Cohen’s *d*	*p*-Value
Stigma [49]	20.39 (6.36)	21.38 (6.74)	0.15	0.084
Operational stress [39,50]	59.54 (21.60)	57.45 (22.63)	−0.10	0.285
Organizational stress [39,50]	55.80 (22.61)	58.72 (24.11)	0.13	0.160
Work engagement [41]	34.64 (8.22)	34.06 (8.16)	−0.07	0.420
PTSD symptoms [52]	16.55 (14.78)	16.15 (15.11)	−0.03	0.764
Anxiety symptoms [53]	5.81 (4.67)	4.68 (4.40)	−0.25	0.005 **
Depression symptoms [54]	5.10 (5.08)	5.16 (5.03)	0.01	0.900
Social interaction phobia [55]	7.49 (8.88)	10.18 (10.33)	0.29	0.002 **
Alcohol use disorder [58]	5.99 (4.90)	4.61 (3.98)	−0.30	<0.001 **
Individual resilience [59]	130.98 (17.02)	126.38 (18.00)	−0.26	0.020 *
Team psychological safety [60]	31.33 (7.26)	35.44 (8.02)	0.53	<0.001 **

Note. * *p* < 0.05, ** *p* < 0.01.

**Table 4:** Pre-post survey score comparison for resilience training.

	Time Point	Sample Size	Metric 1 Mean (SD)	Metric 2 Mean (SD)	Metric 3 Mean (SD)	Total Score Mean (SD)
Individual resilience	T1	131	RSA: 96.66 (7.23)	CSI.E: 22.79 (4.02)	CSI.D: 27.99 (3.40)	—
	T2	15	RSA: 97.00 (3.32)	CSI.E: 23.40 (4.36)	CSI.D: 27.07 (1.87)	—
	T3	18	RSA: 92.61 (15.59)	CSI.E: 22.39 (5.03)	CSI.D: 27.11 (5.88)	—
Family resilience	T1	31	Belief: 43.68 (10.75)	Org.: 32.94 (7.15)	Comm.: 36.45 (9.06)	113.06 (25.80)
	T3	12	Belief: 50.33 (7.76)	Org.: 36.00 (6.09)	Comm.: 40.00 (6.55)	126.33 (18.52)

**Table 5:** Strong associations (Kendall’s τ >0.6 or <−0.6) between organizational survey measures and administrative indicators.

		Extended Health Cost	Sick Leave Hours
T1	Operational stress	0.67	—
	Organizational stress	1.0	—
	Depression	−0.67 *	—
T2	Organizational stress	0.67	—
	Engagement	−0.67	—
	PTSD	0.67	—
	Anxiety	0.67	—
	Depression	0.67	—
	Social phobia	0.67	—
	Resilience	−0.67	—
	Team psych. safety	0.67 *	—
T2 − T1		**Avg. Extended Health Cost**	**Avg. Sick Leave Hours**
	Organizational stress	0.67	—
	Engagement	−0.67	—
	PTSD	0.67	—
	Anxiety	1.00	—
	Depression	0.67	—
	Social phobia	0.67	—
	Resilience	−0.67	—
	Team psych. safety	0.67 *	—

* Indicates results that may be counterintuitive or unexpected.

## Data Availability

Data for this study are not open nor have publicly available archived datasets.
